# The Influence of Perceptions of Competence on Motor Skills and Physical Activity in Middle Childhood: A Test of Mediation

**DOI:** 10.3390/ijerph20095648

**Published:** 2023-04-26

**Authors:** Jeff R. Crane, John T. Foley, Viviene A. Temple

**Affiliations:** 1 School of Human Kinetics and Recreation, Memorial University of Newfoundland, St. John’s, NL A1B 1T5, Canada; 2 Department of Physical Education, The State University of New York at Cortland, Cortland, NY 13045, USA; 3 School of Exercise Science, Physical & Health Education, University of Victoria, Victoria, BC V8P 5C2, Canada

**Keywords:** perceptions, children, motor development, structural equation modelling

## Abstract

The mediating effect of perceptions of physical competence (PPC) on the relationship between motor competence and physical activity levels is documented in adolescence. However, it is unclear at what age this begins. In this study, we examined whether PPC mediated the relationship between moderate–vigorous physical activity (MVPA) or sedentary behaviour and motor competence in middle childhood. The participants were 129 children (mean age = 8.3 years) from eight elementary schools. MVPA and sedentary behaviour were measured using Actigraph accelerometers, and motor competence was assessed using the Test of Gross Motor Development, Second Edition. The Pictorial Scale of Perceived Competence and Social Acceptance for Young Children and The Self-Perception Profile for Children were used to assess PPC. In this study, PPC did not predict either MVPA or engagement in sedentary behaviours. Further, structural equation modelling revealed that PPC did not mediate the relationship between motor competence and MVPA or between motor competence and sedentary behaviour. These results suggest that at 8 years of age, children’s perceptions do not influence their participation in physical activities. It is possible that factors influencing PPC, such as peer comparisons and performance outcomes, have more impact in later childhood or adolescence. In turn, those perceptions may affect children’s or adolescents’ choices to opt in or out of physical activities.

## 1. Introduction

Despite the evidence that physical activity during childhood and adolescence has physical and psychological benefits [1,2], levels of physical activity are typically low and decrease with age, while sedentary behaviours increase [3,4,5,6]. The transition from early to middle childhood is of particular interest, as it is a period when children’s motor and perceptual systems develop rapidly [7,8], their cognitive skills (e.g., memory, attention span, and decision making) become more mature [7,9], and their at-school and after-school lives become more structured and demanding [10,11]. It is likely that these personal and environmental changes interact to affect children’s physical activity levels and patterns [12]. 

The developmental systems perspective posits that developmental outcomes arise from recurring interactions between the individual and his or her environment as well as the integration of individual’s attributes, such as their biology, physiology, motivation, and cognition [8,13]. Lerner and colleagues [13] also argued that to optimize developmental trajectories such as engaging in healthy behaviours (e.g., physical activity), researchers need to identify the attributes of individuals who display those behaviours and ascertain when in the lifespan these processes are at play. 

**Figure 1:**
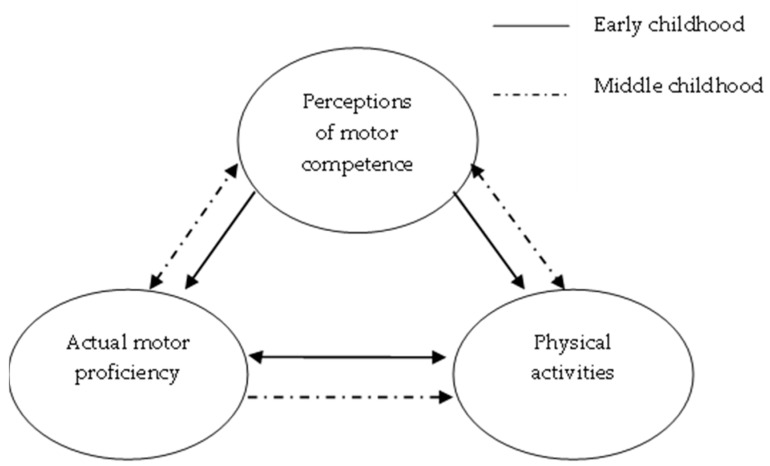
Extract of a model developed by Stodden and colleagues [12] illustrating developmental mechanisms influencing physical activity.

In 2008, Stodden and colleagues [12] published a seminal paper on children’s physical development that identified mechanisms that may influence children’s physical activity participation trajectories (see Figure 1).

As illustrated by this model, motor competence proficiency influences engagement in physical activity directly (mechanism 1) and indirectly via children’s perceptions of their physical competence (mechanism 2).

The first mechanism Stodden and colleagues [12] proposed was that fundamental motor skill proficiency directly influences physical activity levels; however, these authors also suggested that the direction of this influence changes between early and middle childhood. They hypothesized that in early childhood, the relationship is reciprocal [12], with regular bouts of physical activity stimulating neuromotor activity and, therefore, the development of fundamental motor skills, while at the same time, motor skill competence enables participation in activities, games, and/or sports that are physical in nature. Stodden et al. also suggested that by middle childhood, the relationship between motor skill proficiency and physical activity participation will be stronger than in early childhood but unidirectional, so children with higher levels of motor competence will be more physically active in various forms of organized and unorganized physical activities and sports because they have the necessary skills to participate with. Conversely, children who demonstrate lower levels of gross motor proficiency will have lower engagement in physical activity [12]. 

The model (Figure 1) also illustrates a second mechanism that is thought to influence participation in physical activities, and this mechanism is also believed to change from early to middle childhood. Mechanism 2 involves children’s perceptions of their physical competence. As Figure 1 illustrates, perceived physical competence is thought to influence both motor skill development and participation in physical activity in early childhood. Young children tend to have unrealistically high perceptions of their physical competence [14] because they are unable to differentiate between competence and effort or compare their own performances to those of their peers. As a result, young children often rely on feedback from significant others, which is generally very positive [15]. Consequently, children’s beliefs about their physical competence are often inflated or exaggerated. These inaccurate beliefs help children continue to be physically active and persist with activities that nurture motor skill proficiency [12]. Therefore, in early childhood, inaccurate perceptions of physical competence may help in the development of proficient motor skills, and these motor skills support physical activity engagement [16,17,18].

As children transition from early to middle childhood, their perceptions of competence should become reciprocally related to their motor skill proficiency (see Figure 1) as they begin to assess their own physical competence more accurately [12]. This increased accuracy is related to the development of higher cognitive functions [19] such as working memory and cognitive control processes [20]. These enhanced cognitive abilities allow children to be more aware of their own competence levels and performances, compare their performances to their peers’ performances, and analyze the reasons for their successes and failures [15,21]. These more accurate perceptions mean that children with less proficient skills are more likely to have less favourable perceptions of their physical competence [12].

Systematic reviews of the literature have demonstrated that positive perceptions of physical competence are associated with greater physical activity among adolescents [22,23], and lower levels of perceived competence are associated with dropout from organized sports among children and youth [24]. When a child is not succeeding in an activity, they may perceive their competence less favourably and discount the importance of, or withdraw from, physical activities in order to protect their self-esteem [14]. As Stodden and colleagues [12] theorize for middle and late childhood, “…low levels of motor competence will be significantly related to lower perceived motor skill competence and, subsequently, lower levels of physical activity”. 

Although not represented in the Stodden et al. [12] model, sex differences have been identified in physical activity, sedentary behaviour, motor skill proficiency, and perceptions of physical competence, as well as in the interactions between some of these factors. Overall, boys are more active than girls [4,6,25], but there does not appear to be a sex-based difference in sedentary behaviour during early childhood [16] or middle and late childhood [4]. The findings for motor skills are mixed. The literature consistently shows that boys have significantly better object control skills than girls [26,27,28]. However, for locomotor skills, some studies found girls performed better than boys [26,27,29], while other studies showed no differences between boys and girls [30]. Finally, few studies have examined childhood perceived competence levels and sex [27,28,31]. LeGear and colleagues [27] found that 5-year-old girls’ perceived physical competence was higher in comparison to boys, while higher perceived physical competence/ability beliefs were found for 4- [28] and 8-year-old [32] boys compared with girls. Finally, Goodway et al. [31] found no sex-based differences in perceptions of physical competence among 3- and 4-year-olds.

Sedentary behaviour is also not included in the original Stodden et al. [12] model as an outcome variable. Rather, physical activity is the health behaviour variable included in the model, with physical activity precipitating either positive or adverse weight status depending on whether physical activity is high or low, respectively. However, high and low are relative terms. Sedentary behaviour assessed via accelerometry has been previously classified as a lack of moderate-to-vigorous physical activity (MVPA) [33,34], which is not the same as being sedentary. Sedentary behaviours are pursuits that require a low amount of energy expenditure, such as sitting down at a table or using an electronic device [35]. This type of behaviour is characterized by low metabolic equivalents (METs), and these have been operationalized as <1.5 METs [34,35,36]. An absence of MVPA, on the other hand, could mean that slow walking or other forms of light activity (≥1.5 to <4 METs) could be included as “sedentary” behaviours. To understand the relationships between motor skills, perceptions of competence, and participation in activities more fully, both MVPA and sedentary behaviour have been included in the conceptualized model in this study. 

To address a gap between what is known for early childhood and what is known for adolescence, this study examined whether perceptions of physical competence mediated the relationship between motor competence as the predictor variable and both physical activity and sedentary behaviour as dependent variables among children in grades 2 or 3 who were 7 years of age or older. Specifically, the following research questions were addressed: (1) What are the physical activity levels, motor skills, and perceptions of physical competence levels of children? (2) What is the relationship between fundamental motor skills and physical activity and sedentary behaviour? (3) What is the relationship between perceived physical competence and physical activity and sedentary behaviour? (4) What is the relationship between perceptions of competence and motor skill proficiency? (5) Does perceived physical competence mediate the relationship between fundamental motor skill proficiency and physical activity using structural equation modelling?

## 2. Materials and Methods

### 2.1. Design

Structural equation modelling was used in this cross-sectional study to test whether perceived physical competence mediated the relationship between fundamental motor skill proficiency (locomotor and object control) and MVPA and sedentary behaviour. It should be noted that this cross-sectional study is situated within a larger longitudinal study (kindergarten to grade 5), and we have previously published data on grade 2 fundamental motor skills and perceptions of physical competence [37,38], grade 2 physical activity and sedentary behaviour [39], and grade 3 fundamental motor skills [32]. The current study builds on these works by testing the interrelationships between these variables. Descriptive data in this study are provided to provide the basis for the analyses and because meeting the inclusion criteria for this study gives rise to a sample that is not identical to prior studies with fewer variables. 

### 2.2. Participants

The University of Victoria Human Research Ethics Board and the school district granted approval for this study. In Victoria, British Columbia, where the study took place, the participating school district rates of vulnerability relating to readiness were lower than, or equivalent to other school districts in the province (Kershaw, Irwin, Trafford, and Hertzman, 2005). Furthermore, census data showed that families living in this city had a median income equivalent to that of the national median (Statistics Canada, 2019). A two-level consent process was implemented in the current study. Parents/guardians were given the option to consent to the at-school portion of the study that involved the assessment of both fundamental motor skills and perceptions of physical competence. In addition, parents/guardians could allow their child to participate in the physical activity portion of the study that involved measuring levels of physical activity through accelerometry. Data were collected on grade 2 (*n* = 39) and grade 3 (*n* = 90) children during two school years, 2012–2013 and 2013–2014. Inclusion criteria for the present study were (1) children were in grade 2 or grade 3 from one of eight consenting schools, (2) parents consented to the accelerometry portion of the study, (3) the accelerometer wear-time criteria were met, and 4) the child had complete physical activity, fundamental motor skill, and perceived physical competence data. Of the potential *n* = 1.016 grade 2 and 3 children who could have been recruited from the eight schools, parent/guardian informed consent and children’s assent were obtained for *n* = 705 children. Of the recruited children, 198 parents/guardians consented to the accelerometry portion of the study, and *n* = 129 met inclusion criteria (3) and (4). Although this study was part of a longitudinal study, no child was included twice in this sample. If a child had complete data in both grades 2 and 3, grade 3 data were prioritized.

### 2.3. Measures

The Test of Gross Motor Development, Second Edition [TGMD-2] [40] was used to assess the fundamental motor skill competence of participants. The TGMD-2 is a criterion and norm-referenced test that measures locomotor and object control skills. Specifically, the 12-item tool measures 6 locomotor skills (running, jumping, hopping, sliding, galloping, and leaping) and 6 object control skills (throwing, rolling, kicking, striking, catching, and dribbling). The TGMD-2 has established content validity, predictive validity, and construct validity (Ulrich, 2000), as well as predictive validity for children aged 3–10 years. Reliability of the TGMD-2 was established by examination of scale internal consistency scores, test-retest reliability, and inter-scorer differences [40]. 

Physical activity was measured using Actigraph GT1M accelerometers (ActiGraph, LLC, Fort Walton Beach, FL, USA). The GT1M is an electronic device that measures and records acceleration or movement. Pre-filtered data are collected by the uni-axial device at a rate of 30 measurements per second (30 Hz) and then post-filtered into measurements known as epochs. The accelerometer is worn around the waist, with the device positioned on the right hip. For the present study, 15 s epochs were used to record physical activity, as suggested by Reilly and colleagues, in order to record the sporadic activity of children [41] before being converted to a metabolic equivalent (MET). The Actigraph accelerometer has been shown to be a valid indicator of energy expenditure and activity levels in children and youth [42,43].

The Pictorial Scale of Perceived Competence and Social Acceptance for Young Children [44] and the Self-Perception Profile for Children (SPPC) [45] were used to assess perceived physical competence (PPC) for grade 2 and grade 3 children, respectively. The pictorial scale has demonstrated both reliability and validity for grade 1 and grade 2 children [44]. Internal consistency reliability was established for grade 1 and grade 2 children at 0.86 for total scale and 0.55 for the physical domain. Further, convergent validity was determined in the physical domain, as children could provide concrete reasons for their competency. The 24-item scale is divided into 4 subscales; however, for the purposes of this study, only the PPC sub-scale was used. Administration of the scale was in the form of bipolar statements that are supplemented by a picture. Each item was scored on a four-point scale, where a 4 indicates the highest degree of perceived competence and 1 the lowest. 

For grade 3 participants, the Self-Perception Profile for Children has demonstrated both acceptable reliability and validity for children 8 years or older [46]. Subscale reliability for the physical domain was 0.83. Further, convergent validity was determined in the physical domain by correlations between the physical educator’s rating of the students’ physical competence and the students’ ratings of their physical competence. The scale consists of 36 items subdivided into a global self-worth scale and 5 subscales (6 statements each) that include Scholastic Competence, Social Competence, Athletic Acceptance, Physical Acceptance, and Behavioural Acceptance. For the purposes of this study, only the perceptions of physical competence sub-scale were used. Each item in the subscale was phrased to the child in a “structure alternative format”. For example, children were given two statements and asked to choose which statement more closely resembled them (e.g., “Some kids do very well at all kinds of sports” BUT “Other kids don’t feel that they are very good when it comes to sports”). Once the child selected the statement that resembled them, a second question was asked to determine the level of competence (i.e., “Is that ‘really true for me’ or ‘sort of true for me’”). In accordance with the previous scale, each item is scored on a four-point scale, where a 4 indicates the highest degree of perceived competence and 1 the lowest.

For younger children in this study, including the pictorial format is a developmentally appropriate measure, as it helps them better understand the question, which supports accuracy in responses. For older children, we included more general descriptions with developmentally appropriate language. The underlying construct examined by both measures in this study was children’s perceptions of their physical competence. Susan Harter developed both of these measures to examine this construct in a developmentally appropriate way [44].

### 2.4. Procedures

Accelerometers were initialized to record physical activity on the first day of motor skill testing. Each accelerometer was placed on the child immediately following administration of the TGMD-2. The accelerometer was worn for seven days from when the child first woke up in the morning until they went to sleep, unless they were bathing or swimming. In accordance with previous studies with young children, the minimum wear time criteria to be considered a valid physical activity record was 10 h per day for at least four days, which includes at least three weekdays and one weekend day [47].

The TGMD-2 was administered during scheduled physical education class in accordance with the testing procedure outlined in the Examiner’s Manual [40]. A research team consisting of 10 research assistants and 3 program coordinators was used throughout the data collection. Children were digitally recorded performing two trials of each skill at their station before rotating to the next station. Perceived physical competence was measured with children by a trained administrator. Consenting children were removed from the classroom and taken to a quiet location in the school where there was minimal noise and distraction (e.g., an available multi-purpose room).

### 2.5. Data Treatment

The investigators scored the behavioural components of the locomotor skills and object control skills for each child from the digital video. Each child was given a raw score out of a possible 48, which corresponds to the number of components completed correctly for each subtest. The inter-observer reliability was established by two investigators re-scoring 15% of the digital videos for each of the classes [27,48]. Percent agreement in the present study reported a mean of 89.4%. These raw scores were then converted into subtest standard scores (out of 20), to allow for comparisons across ages. Similarly, the items of both perceived competence scales assessing the physical domain were scored on a scale of 1–4 for each item. Raw scores (out of 24) were obtained by summing the 6 items from the physical competence scale.

Raw data from the accelerometers were downloaded using ActiLife software version 6 (Actigraph LLC) for subsequent data reduction. Kinesoft software (version 2.0.94, Kinesoft Software, New Brunswick, Canada) was used to inform the analysis, as well as for extraction and processing of the physical activity data. Physical activity and sedentary behaviour were classified into the following intensities based on previous studies with children [17,36,49]: sedentary behaviour < 1.5 METs and moderate-to-vigorous physical activity (MVPA) ≥ 4.0 METs.

### 2.6. Statistical Analyses

Descriptive statistics were computed for raw scores and standard scores for both subscales of the TGMD-2 skills, raw scores for perceived physical competence, average minutes of physical activity per day (MVPA and total physical activity), and sedentary behaviour using SPSS^®^ 23 for Windows (IBM Corp, 2015).

Structural equation modelling (SEM) was used in the present study to examine mediation. SEM uses a conceptual model and path diagram to identify complex and dynamic relationships between observed and unobserved variables [50]. In comparison to using a regression model, SEM simplifies testing mediation because of its ability to test these more complicated mediation models in a single analysis. Further, SEM analyses provide model-fit information about the consistency of the hypothesized model to the data [50]. Path diagrams are typically used to represent SEM models. In the present study, the path analysis was conducted using Mplus (Version 6.12) [51] to investigate whether perceived physical competence mediated the relationship between fundamental motor skill proficiency (locomotor and object control) and MVPA and/or sedentary behaviour. Specifically, the path analysis examined the directional relationships between model variables directly and indirectly. Due to the complexity of path analysis, the process consisted of multiple stages: (1) Constructing a conceptual model based on theory and the literature; (2) Identifying direct and indirect paths for both dependent and independent variables; (3) Testing the model to determine that it was of good fit; (4) Reporting findings, fit indices, and results from model; (5) Re-specifying the conceptual model if warranted and reporting findings, fit indices, and results from the re-conceptualized model.

### 2.7. Constructing a Conceptual Model

**Figure 2:**
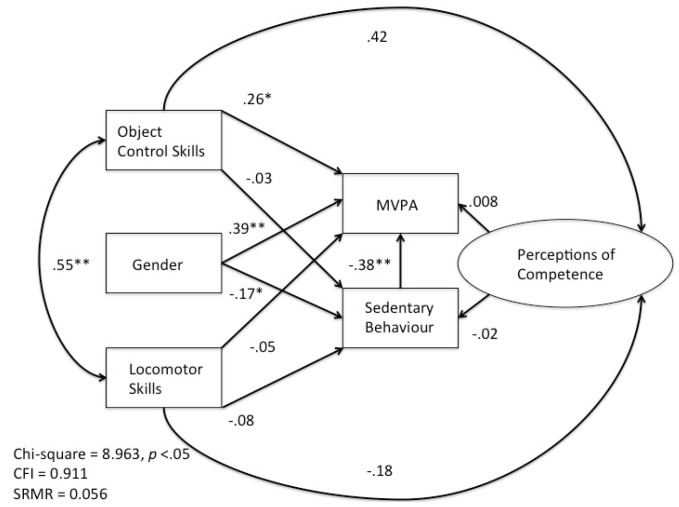
Path model depicting the relationships in a new model of developmental trajectories (*n* = 129) * *p* < 0.05 ** *p* < 0.001.

The extract of Stodden and colleagues’ [12] theoretical model illustrated in Figure 1 formed the basis of the model for this study. However, the original model did not include sex or sedentary behaviour as variables. Therefore, based on known sex-based differences in motor skill proficiency, perceptions of competence, and physical activity levels described in this paper’s introduction, a new conceptualized model was developed (see Figure 2) with directional arrows to model the relationships between the variables and to test whether perceived physical competence mediated the relationship between motor competence and MVPA and/or sedentary behaviour. 

#### 2.7.1. Identifying the Directional Arrows for the Proposed New Model

Specifically, the new model (Figure 2) examined (1) the relationships between locomotor and object control skills separately with MVPA, sedentary behaviour, and perceived physical competence; (2) the relationship between perceived physical competence and MVPA and sedentary behaviour; (3) the indirect influence that perceived physical competence had on the relationship between motor competence and MVPA; and (4) the relationship between sex and both MVPA and sedentary behaviour.

#### 2.7.2. Testing the Proposed Model

A structural model was tested using a covariance matrix as input and maximum likelihood estimation. This analysis provided a comprehensive depiction of the associations between the predictor and dependent variables of interest. The structural model consisted of three observed independent variables (locomotor skills, object control skills, and sex) and three observed dependent variables (perceived physical competence, MVPA, and sedentary behaviour). In determining the overall fit of structural equation model analyses, the chi-square test, Comparative Fit Index (CFI), and Standardized Root Mean Square Residual (SRMR) were used to assess how well the model fit the data [52]. The CFI values should be between 0 and 1, with values more than 0.95 considered to be a good fit for the data [52,53]. For the SRMR, a value of less than 0.05 indicates a close fit [52,53]. An SRMR of 0 indicates perfect fit, but it must be noted that SRMR will be lower when there is a high number of parameters in the model and in models based on large sample sizes.

## 3. Results

A total of 129 participants (mean age = 8 years 3 months, 52% boys) met the wear-time criteria for accelerometry and had their motor competence and perceived physical competence measured in the present study. Descriptive statistics for all participants and by sex for fundamental motor skills, perceived physical competence, MVPA, and sedentary behaviour are reported in Table 1.

The locomotor and object control skill scores were very similar, and participants scored in the middle range. Additionally, perceptions of physical competence were generally positive and physical activity levels were high. Children in this study achieved an average of 30 min more than the Canadian recommendation of at least 60 min of MVPA per day [54] and accumulated approximately 460 min of sedentary behaviour per day.

### Path Model Analysis for the Specified Model

The results of the path analysis with the standardized coefficients for each variable are presented in Figure 2. 

**Figure 3:**
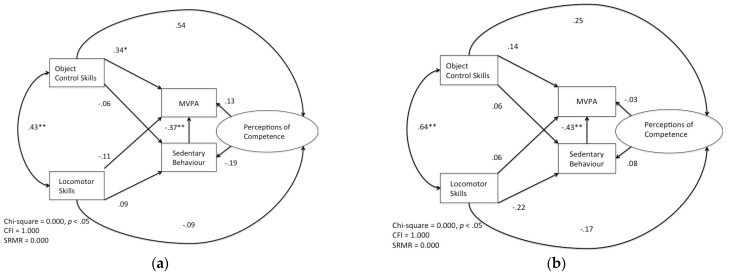
(**a**) The re-specified path model depicting the relationships of developmental trajectories for boys (*n* = 67) * *p* < 0.05 ** *p* < 0.001. (**b**) The re-specified path model depicting the relationships of developmental trajectories for girls (*n* = 62) *p* < 0.05 ** *p* < 0.001.

The model satisfied the chi-square specifications χ^2^ = 8.963 (*df* = 2, *p* < 0.05); however, it did not satisfy the fit indices for either the CFI = 0.911 or SRMR = 0.056. This suggests that the overall fit of the model is relatively poor. Based on these results, the model needed to be re-conceptualized and re-specified. Sex was removed from the model on the basis that for each variable measured, researchers have continuously shown that the strength of the relationships that exist among these variables is different for boys and girls. Therefore, in re-specifying the model, two identical models were created for boys (Figure 3a,b) and for girls, which became the final specified models in this study.

The re-specified model for boys displayed an overall good fit. The chi-square specifications χ^2^ = 0.000 (*df* = 0, *p* < 0.001) and the CFI = 1.000 and SRMR = 0.000 suggest that the re-specified model has an excellent fit. The results from model 1 suggest that object control skills have a significant direct effect on MVPA (β = 2.980, SE = 1.220, β = 0.341, *p* < 0.05). Furthermore, sedentary behaviour (β = −0.173, SE = 0.071, β = −0.368, *p* < 0.001) directly and negatively affected MVPA. Finally, there was also a significant relationship between locomotor and object control skills (β = 1.866, SE = 0.580, β = 0.431, *p* < 0.001). However, no other direct effects or relationships were established through the path analysis. The indirect effect was tested using bootstrapping procedures. For MVPA, the bootstrapped unstandardized indirect effect was 0.028 and the 95% confidence interval (CI) was [−2.372, 2.428], suggesting that the indirect effect was not significant. For sedentary behaviour, the bootstrapped unstandardized indirect effect was −0.086, and the 95% CI was [−3.381, 3.209], which suggests there was not an indirect effect. 

The second specified model for girls also displayed an excellent overall fit. The chi-square specifications χ^2^ = 0.000 (*df* = 0, *p* < 0.001) and the CFI = 1.000 and SRMR = 0.000 demonstrate that all fit indices were satisfied. The results from the model suggest that sedentary behaviour (β = −0.136, SE = 0.035, β = −0.426, *p* < 0.001) has a direct negative effect on MVPA. There was also a positive and significant relationship between locomotor and object control skills (β = 3.532, SE = 0.987, β = 0.641, *p* < 0.001). However, no other direct effects or relationships were established through the path analysis. The indirect effect was tested using bootstrapping procedures. For MVPA, the bootstrapped unstandardized indirect effect was −0.029, and the 95% CI was [−1.042, 0.985], suggesting the indirect effect was not significant. For sedentary behaviour, the bootstrapped unstandardized indirect effect was 0.051, and the 95% was [−4.154, 4.256], which suggests there was not an indirect effect.

## 4. Discussion

This study examined whether PPC mediated the relationship between motor competence and physical activity and/or sedentary behaviour in middle childhood. The three indices (chi-square, CFI, and SRMR) used to measure the fit of the data [52,53] demonstrated that the first hypothesized model did not fit the data statistically. As a result, the model was re-specified as two separate sex-based models, which had an excellent fit. 

The most important finding, which was demonstrated for both boys and girls, was that perceived physical competence did not mediate the relationship between motor skill proficiency and MVPA or between motor skill proficiency and sedentary behaviour. Additionally, perceptions of competence did not predict either MVPA or engagement in sedentary behaviours. These findings were unexpected but add to our understanding of the role of perceptions of physical competence from a developmental perspective. Developmental theorists have suggested [15], and recent evidence supports [38], that as children transition to middle childhood, the accuracy of their perceptions should start to improve. These enhanced cognitive abilities allow children to be more aware of their own competence and performances [55], compare their performances to their peers’ performances, analyze the reasons for their successes and failures, and internalize the feedback they receive [15,21,56]. Logically, then, more developed motor skills lead to more positive self-perceptions, which, in turn, are associated with higher engagement in physical activities. However, this pattern, which was established among adolescents, [26] was not evident in these data. Most of the children in this study were in grade 3, and these grade 3 children had lower PPC compared to their grade 2 peers. While it appears that self-perceptions are lower and perhaps becoming more realistic in grade 3, these higher positive self-perceptions observed in grade 2 may have influenced our findings. As children become more independent of their parents in their choices of active and inactive play during the transition from early to middle childhood [57], it is also possible that there is a lag time between children’s changing perceptions and their activity choices that are likely negotiated with their parents. 

Another consistent finding of both models was that sedentary behaviour was a significant negative predictor of MVPA. The negative direct relationship between sedentary behaviour and MVPA shows that both boys and girls who are engaging in higher levels of sedentary pursuits are also spending less time in MVPA. Some children in this study were spending as little as 30 min engaged in MVPA per day and as much as 10 h per day sedentary. These findings contrast the “active couch potato” trend previously used to identify children who simultaneously meet daily physical activity guidelines while still spending the majority of the day sedentary [35]. This study shows that children who are highly sedentary are also achieving low levels of MVPA per day, which is concerning because of the relationship between sedentary behaviours, metabolic syndrome, and hypertension [35,58,59]. Continued efforts need to be made to reduce the amount of time spent sedentary and increase physical activity levels. 

The relationships between motor skill proficiency and MVPA and motor skill proficiency and sedentary behaviour were vastly different for boys and girls in this study. For boys, object control skills significantly predicted MVPA levels. This positive relationship was consistent with previous evidence examining this relationship [17,26]. Interestingly, although locomotor skills and object control skills were related in this study, locomotor skills did not predict MVPA. Barnett and colleagues’ [26] study of mediation also found that while locomotor skills and object control skills were related, their model of mediation included object control skills as a predictor of physical activity. Additionally, neither locomotor skills nor object control skills were related to sedentary behaviour. To date, only Wrotniak and colleagues [60] have examined the relationship between motor skill proficiency and sedentary behaviour in middle childhood. Wrotniak et al. found that the relationship between sedentary behaviour and fine and gross motor skills was negative. Although there is only a modicum of evidence, it appears that while object control skills predict the MVPA of boys, motor skill proficiency does not affect time spent being sedentary at this age. 

For girls, although the relationship between locomotor skills and object control skills was significant and positive, motor competence was not associated with MVPA or sedentary behaviour. These findings are somewhat difficult to explain. Cliff and colleagues [16] suggested that these relationships are absent or negative for girls because it is possible that the skills they typically develop are not properly represented in motor skills assessments. This thought is supported to some degree by Temple and colleagues [61], who, after finding no significant relationships between girls’ motor skills and questionnaire-based physical activities, found that the leap was significantly correlated with girls’ participation in both physical activities and active physical recreation. This finding may suggest that while the physical activities girls are currently participating in help in developing motor competence levels, the activities do not contribute to MVPA such as running around on a soccer field. Therefore, additional research about the best approach to measure girls’ skillfulness is needed.

Although it is possible that the TGMD-2 does not assess the motor skills used by girls when they are active, it is also possible that factors other than the ones included in the model are far more important predictors of girls’ participation. It is also possible that the girls with better motor skills do not perceive themselves as skillful or that less skillful girls do not perceive themselves as less physically competent. This was evident in Field et al.’s [38] longitudinal study of the trajectory of PPC accuracy among children from grade 2 to grade 4. These authors found that there was a proportion of boys and girls in grade 2 who underestimated their motor competence. A particularly troubling finding was that the underestimating boys increased their PPC by grade 4 to have a more realistic self-appraisal, but the underestimating girls’ PPC did not change across that two-year period and their motor competence stagnated. It is clear from our study that the re-specified model did not explain girls’ participation in MVPA or sedentary behaviour and that more research is needed to identify factors influencing girls’ participation in middle childhood. Emadirad et al.’s [32] finding that it was how much grade 3 girls valued physical activity, not ability beliefs or expectations of success, that mediated the relationship between motor competence and physical activity, may help point the way. 

A limitation of the current study was the loss of participants for whom valid accelerometer data were available. The accelerometer wear-time criterion was 600 min of wear time per day for three weekdays and one weekend day. This resulted in 35% of participants being lost. Parents and guardians also indicated that children often forgot to wear the accelerometer or would take it off due to comfort issues. There is also a possibility that parents and guardians who consented may be more engaged in physical activity than families who did not consent in the present study, which may have had an impact on physical activity levels among participants. 

## 5. Conclusions

PPC did not mediate the relationship between motor competence and physical activity or sedentary behaviour in middle childhood for boys or for girls. Overall, the participants could be characterized as having high physical activity and sedentary behaviour levels and high PPC. However, their actual motor competence, as assessed by the TGMD-2 was quite low for their age. The relative levels (i.e., high or low) for each variable in the specified models may in part explain the findings. It might be expected that, at grades 2/3, children’s perceptions of physical competence would start to become more accurate (and thereby lower) than when they were younger. However, the findings of this study revealed that perceptions of physical competence were quite high. These findings illustrate that factors other than motor competence are influencing children’s PPC in middle childhood and that children’s perceptions of their abilities are not influencing their participation. It is possible that children are not using peer comparisons and performance outcomes to form their perceptions at this age, but it is also possible that other factors (e.g., parents enrolling their children in sports) are more powerful influences on participation.

The negative relationship between MVPA and sedentary time in both models in this study is notable. Unfortunately, boys and girls with higher sedentary time were also more likely to have low levels of MVPA. Like most research examining the relationship between motor skills and physical activity among boys, object control skills predicted MVPA levels. It appears that these are the “tools” being used by boys when they are active. The complete absence of significant paths between motor skills (locomotor or object control) and MVPA among girls is somewhat difficult to explain, but as noted above, may be related to the skills assessed. It is also possible that there are other influences on girls’ participation, or both dynamics may be at play. What is clear is that the re-specified model does not explain girls’ participation in MVPA or sedentary time and that more exploratory work is needed to identify more salient factors.

## Figures and Tables

**Table 1:** Descriptive statistics for motor skills, perceived physical competence (PPC), physical activity (MVPA), and sedentary behaviour.

		All Children *n* = 129				Boys *n* = 67		Girls *n* = 62	
Variable	Range	Mean	SD	Min	Max	Mean	SD	Mean	SD
Locomotor skills raw scores	0–48	32.0	5.3	13.0	44.0	31.4	5.6	32.4	5.1
Object control skills raw scores	0–48	32.8	6.7	17.0	46.0	36.3	6.4	29.5	5.2
Locomotor standard scores	0–20	5.6	2.1	1.0	16.0	5.2	1.6	6.2	2.2
Object control standard scores	0–20	6.0	1.6	1.0	13.0	5.8	2.7	6.2	2.5
PPC	6–24	18.6	3.7	9.0	24.0	18.2	3.5	18.9	3.1
MVPA	min/day	92.6	24.7	37.9	170.9	103.9	23.3	82.2	21.2
Sedentary behaviour	min/day	463.7	53.9	339.0	642.0	454.5	49.6	472.2	66.4

## Data Availability

The data presented in this study are available on reasonable request from the corresponding author. The data are not publicly available due to confidential personal details and health information.
